# A new method for quantifying glyoxalase II activity in biological samples

**DOI:** 10.1093/biomethods/bpae069

**Published:** 2024-09-18

**Authors:** Mohammed Alaa Kadhum, Mahmoud Hussein Hadwan

**Affiliations:** Department of Chemistry, College of Science, University of Babylon, Hillah City 51002, Iraq; Department of Chemistry, College of Science, University of Babylon, Hillah City 51002, Iraq

**Keywords:** glyoxalase system, DNPH method, medical application, methylglyoxal, Bland–Altman plot

## Abstract

Glyoxalase II (Glo II) is a crucial enzyme in the glyoxalase system, and plays a vital role in detoxifying harmful metabolites and maintaining cellular redox balance. Dysregulation of Glo II has been linked to various health conditions, including cancer and diabetes. This study introduces a novel method using 2,4-dinitrophenylhydrazine (2,4-DNPH) to measure Glo II activity. The principle behind this approach is the formation of a colored hydrazone complex between 2,4-DNPH and pyruvate produced by the Glo II-catalyzed reaction. Glo II catalyzes the hydrolysis of S-D-lactoylglutathione (SLG), generating D-lactate and reduced glutathione (GSH). The D-lactate is then converted to pyruvate by lactate dehydrogenase, then reacting with 2,4-DNPH to form a brown-colored hydrazone product. The absorbance of this complex, measured at 430 nm, allows for the quantification of Glo II activity. The study rigorously validates the 2,4-DNPH method, demonstrating its stability, sensitivity, linearity, and resistance to interference from various biochemical substances. Compared to the existing UV method, this 2,4-DNPH-Glo II assay shows a strong correlation. The new protocol for measuring Glo II activity using 2,4-DNPH is simple, cost-effective, and accurate, making it a valuable tool for researchers and medical professionals. Its potential for widespread use in various laboratory settings, from academic research to clinical diagnostics, offers significant opportunities for future research and medical applications.

## Graphical Abstract

**Graphical Abstract:**
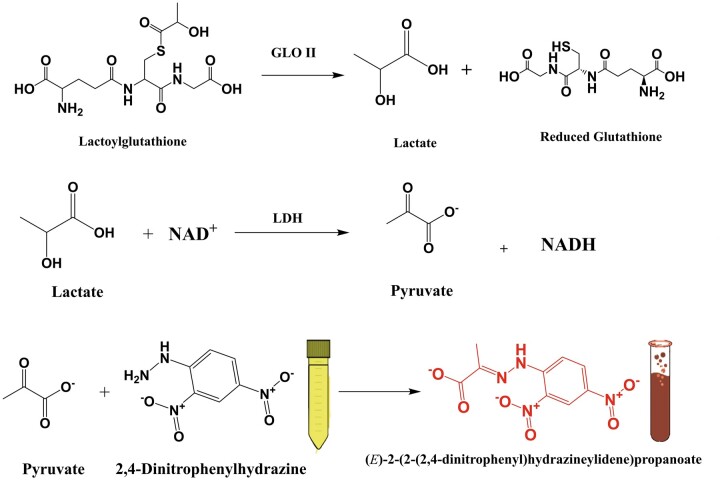

## Introduction

Glyoxalase II (Glo II) is an enzyme that plays a role in multiple regulatory pathways and is involved in the detoxification of dicarbonyl metabolites such as methylglyoxal (MGO) and glyoxal (GO) [1]. It works with glyoxalase I (Glo I) to maintain low levels of these toxic metabolites [2].

Glo II is a pivotal factor in cellular redox balance and various metabolic processes, playing a significant role in diseases like cancer, diabetes, and vascular complications [3, 4]. Its detoxification of MGO by catalyzing the hydrolysis of SLG as a crucial activity in the glyoxalase system demonstrates its antioxidant properties [4]. The dysregulation of the glyoxalase system, including Glo II, is implicated in cancer aggressiveness, drug resistance, and intricate crosstalk with signaling pathways such as MEK/ERK/SMAD1, highlighting its relevance in cancer diagnostics and treatment strategies [4]. Recent studies have revealed additional roles for Glo II, particularly in oxidative-stress-related diseases such as cancer. Glo II’s involvement in oxidative stress-related diseases and its interactions with specific metabolic pathways underscore its emerging roles beyond MGO detoxification, suggesting its potential importance in various pathological conditions [5]. The regulation of Glo II is of significant interest for pharmacological interventions aimed at combating aging-related diseases and cancer treatment. In vivo studies using zebrafish have shown that Glo II regulates cellular energy metabolism in the liver and skeletal muscle, impacting glucose homeostasis and postprandial kinase activation [6]. However, understanding the functions and regulatory mechanisms of the glyoxalase system is essential for developing targeted therapies for diseases associated with dicarbonyl stress and oxidative damage. This understanding can significantly impact disease-treatment strategies, particularly in developing new therapeutic targets [6, 7].

The activity of Glo II has historically been determined by quantifying the initial rate at which it hydrolyzes SLG in the presence of a biological sample. This can be achieved by spectrophotometrically monitoring the decrease in absorbance at 240 nm, using a molar absorptivity (ε240) value of 3.10 mM^−1 ^cm^−1^. The initial concentration of SLG is set at 0.3 mM in a 50 mM Tris/HCl buffer with a pH of 7.4. The activity of Glo II is reported in units per milliliter of packed red blood cells, where one unit is defined as the amount of enzyme needed to catalyze the hydrolysis of 1 micromole of SLG per minute under the specified assay conditions [8]. Modified protocols that use a UV-transparent microplate for higher sample throughput are also available [9]. The above protocol is generally sensitive and can detect changes in absorbance even at low concentrations, allowing for the detection of small changes in Glo II activity. The measurement of absorbance at 240 nm (A_240_) specifically targets the enzymatic activity of Glo II. This ensures that the measured signal directly relates to the enzyme’s function and provides a specific assessment of its activity. The disadvantage of the protocol includes the possible presence of substances present in the sample that may absorb light at 240 nm, leading to interference with the measurement. This can result in inaccurate readings and affect the assessment of Glo II activity. Changes in A_240_ may not solely reflect changes in Glo II activity. Other factors, such as sample impurities or non-specific reactions, can also contribute to changes in absorbance at this wavelength. Therefore, it is essential to consider potential confounding factors when interpreting the results of assays of the above type.

An alternative and widely used spectrophotometric method for measuring Glo II activity uses 5,5'-dithiobis(2-nitrobenzoic acid) (DTNB), also known as Ellman’s reagent [10]. The DTNB assay for the activity of Glo II is founded on the principle that the enzyme catalyzes the hydrolysis of SLG to produce D-lactate and GSH. The free thiol group (-SH) of the released GSH then reacts with DTNB, producing 2-nitro-5-thiobenzoate (TNB), a yellow-colored compound. The rate of TNB formation, measured by the increase in absorbance at 412 nm, is directly proportional to the Glo II activity in the sample. The assay involves preparing a reaction mixture containing necessary components such as potassium phosphate buffer, DTNB, and SLG (the Glo II substrate). The enzyme-containing sample is then added to initiate the reaction, and the absorbance at 412 nm is measured at regular intervals to determine the change in absorbance per minute (ΔA/min). The Glo II activity is then calculated using a formula that considers the molar extinction coefficient of TNB.

The DTNB assay for measuring Glo II activity is known for its sensitivity and simplicity and has earned widespread use. Its high sensitivity instills confidence in researchers and technicians about the accuracy of their results. This method is relatively straightforward and can be easily adopted in various laboratory settings. Additionally, it is suitable for high-throughput screening and assay of Glo II activity. However, it should be noted that the assay may be susceptible to interference from other thiol-containing compounds in the sample, which could influence the measured Glo II activity. In such cases, additional sample preparation or control experiments may be necessary to ensure the accuracy of the results.

This study presents a novel and innovative method for measuring the activity of Glo II using 2,4-dinitrophenylhydrazine (2,4-DNPH) as a chromogenic reagent. The underlying principle of this approach is the formation of an intensely colored hydrazone complex between 2,4-DNPH and the pyruvate generated from the Glo II-catalyzed reaction. Specifically, Glo II catalyzes the hydrolysis of SLG, producing D-lactate and reduced GSH as byproducts. Subsequently, the D-lactate is rapidly converted to pyruvate by the enzyme lactate dehydrogenase. The pyruvate then reacts with 2,4-DNPH to form a stable, brown-colored hydrazone product, which exhibits a characteristic absorbance maximum at 430 nm. By measuring the absorbance of this complex at 430 nm, the activity of Glo II can be accurately quantified, providing a sensitive and reliable means of detecting and monitoring Glo II enzyme activity.

## Procedure

### Chemicals

Lactoglutathione [(SLG, C_13_H_21_N_3_O_8_S), Molecular Weight: 379.39, CAS Number: 54398-03-7], Sodium phosphate dibasic [(Na_2_HPO_4_), Molecular Weight: 141.96, CAS No.: 7558-79-4], Potassium phosphate monobasic[(KH_2_PO_4_), Molecular Weight: 136.09, CAS No.: 7778-77-0], Nicotinamide adenine dinucleotide hydrate (NAD^+^) [(C_21_H_27_N_7_O_14_P_2_), Molecular Weight: 663.43 (anhydrous basis), CAS Number: 53-84-9], 2,4-DNPH [(H2C=NNHC6H3(NO2)2), Molecular Weight: 210.15, CAS No.: 1081-15-8, Hydrochloric acid [Molecular Weight: 36.46, CAS No.: 7647-01-0], Sulfuric acid [Molecular Weight: 98.08, CAS Number: 7664-93-9], Sodium pyruvate [(CH_3_COCOONa), Molecular Weight: 110.04, CAS No.: 113-24-6], Sodium hydroxide [Molecular Weight: 40.00, CAS No.: 1310-73-2], Ethanol [(CH_3_CH_2_OH) Molecular Weight: 46.07, CAS No.: 64-17-5]. Guanidine hydrochloride [NH_2_C(=NH)NH_2_ · HCl, CAS No.: 50-01-1, Molecular Weight: 95.53 EC No.: 200-002-3]. All chemicals are purchased from Sigma-Aldrich.

### Reagents

For the Tris–HCl buffer solution (200 mM, pH 8), 2.42 g of Tris powder was dissolved in 80 ml of distilled water. Then, 0.43 ml of hydrochloric acid (12.1 M) was added to the solution. The final pH of the solution was adjusted using HCl or NaOH, and more distilled water was added until the total volume reached 0.1 l. Lactic Dehydrogenase working solution consists of lactate dehydrogenase enzyme (150U) and NAD^+^ (33 mg; 1 mM) that are dissolved in 50 ml of Tris–HCl buffer solution (200 mM, pH 8). An excess of this solution can also be made, aliquoted, and stored at −20°C. Lactate dehydrogenase was purified and prepared as described by Ryan [11]. The final activity was 5 U/ml of phosphate buffer (0.1 M, pH 7.4). Tris–HCl buffer solution (100 mM, pH 7.4) was prepared by dissolving 1.21 g of Tris powder in 80 ml of distilled water. Then, 0.31 ml of hydrochloric acid (12.1 M) was added to the solution. The final pH of the solution was adjusted using HCl or NaOH, and more distilled water was added until the total volume reached 0.1 l. Lactoglutathione solution (6.59 mM) was prepared by dissolving 25 mg of SLG in 10 ml of a 100 mM Tris–HCl solution (pH 7.4). A pyruvate standard (5 mM) was prepared by dissolving 55 mg of sodium pyruvate in 100 ml of phosphate buffer (pH 7.4). The working standard was stored in small aliquots in a freezer; one aliquot may be used to prepare a calibration graph. The color reagent was prepared by dissolving 50 mg of 2,4-DNPH in 100 ml of 1 N HCL and storing it in a brown bottle. Sodium hydroxide 1M was prepared by dissolving 4 g of NaOH in 100 ml of distilled water.

### Source of Glo II

Tissue samples were obtained from the spinal cords of 24-week-old male albino rats and stored at −80°C until they were analyzed. Tissue homogenates were prepared using a modified version of the method described by Skapare *et al*. [12]. Specifically, the tissue was homogenized in an ice-cold 10 mM sodium phosphate buffer with a pH of 7.4, using an ultrasonic processor (Ultrasonic Probe Sonicator 20 kHz, BIOBASE, China) set at 20 kHz for 30 s. The weight/volume ratio for homogenization was 1:10. Following homogenization, the mixture was centrifuged at 20,000 g for 10 min at +4°C. The supernatant obtained after centrifugation was used to measure Glo II activity. The protein concentration in the supernatant was measured using the Lowry assay [13].

### Protocol

The Glo II enzyme activity procedure is explained in detail in Table 1.

**Table 1.** Detailed protocol for measuring Glo II enzyme activity.

Reagents	Test	Control	STD	Blank
Tris/HCl buffer (pH 7.4)	500µl	500µl	500µl	500µl
Lactoglutathione solution	100µl	100µl	100µl	100µl
Distilled water	350 µl	350 µl	300 µl	400 µl
Pyruvate	–	–	100µl	–
Trichloroacetic acid	–	100µl	100µl	100µl
The reaction was initiated by adding serum:				
Serum	50 µl	50 µl	–	–
The test tube was mixed by vortexing and then incubated for 10 min at 37°C. Afterward, the enzymatic reaction in the test tube was terminated by adding 0.1 ml of 8% (v/v) TCA, followed by centrifugation for 10 min at 3000 *g*. Subsequently, 0.5 ml of the supernatant was removed and transferred to a clean tube, and then 0.5 ml of 0.1 M Tris–HCl buffer (pH 7.4) was added to neutralize the TCA. The resulting solution was then used for further analysis:				
Lactate dehydrogenase (LDH) working solution	0.5 ml	0.5 ml	0.5 ml	0.5 ml
The test tubes were mixed by vortexing and then incubated for 10 min at 37°C. The LDH enzymatic reaction in the test tubes was then terminated by adding 0.5 ml of DNPH. The solutions were further incubated for 20 min at 37°C. Finally, 1.5 ml of 1 N NaOH was added to each test tube.				

### Calculation

The enzyme activity is measured in International Units (IU), with one IU representing the amount of enzyme needed to catalyze the formation of 1 micromole of pyruvate per minute per liter of serum or supernatant at 37°C. The following equation was applied to calculate Glo II activity in each test tube:

$$\text{Glo}\;\text{II}\;\text{activity}\;\left(U/L\right)=\left(\frac{A\;\mathrm{Test}-A\;\mathrm{Control}}{A\;\text{STD}}\right)\times \text{Conc}.\;\text{of}\;\text{STD}$$

**Figure 1:**
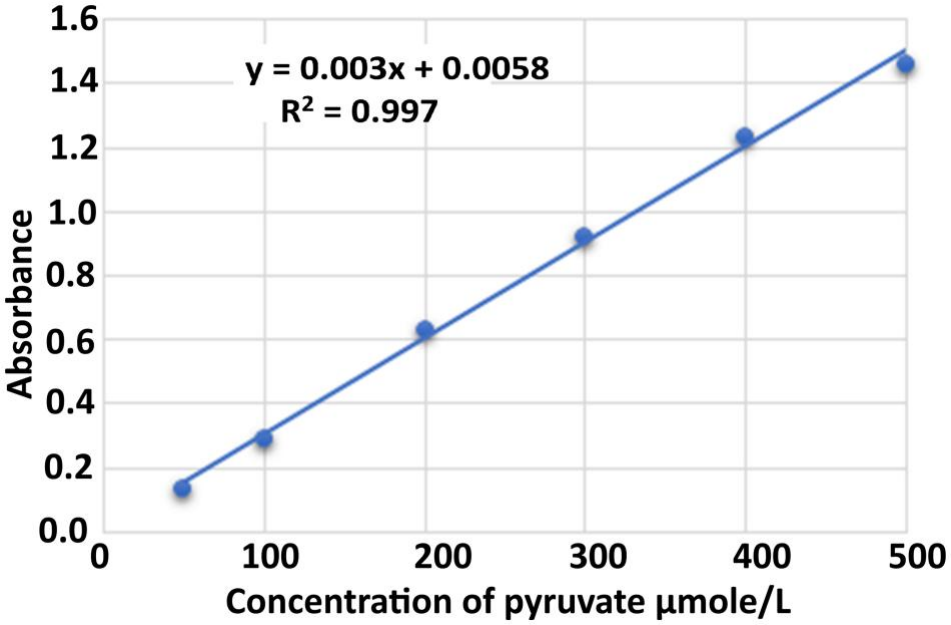
Pyruvate standard curve for the DNPH-Glo II method.

The concentration of the standard (STD) is equal to 500 µM. Additionally, the activity of Glo II can be determined from the standard curve generated using pyruvate (Fig. 1). The method’s standard curve was constructed following the procedure described above, utilizing a pyruvate stock solution. The absorbance of the pyruvate solution was measured at 430 nm using the DNPH-Glo II method. Figure 1 presents a graph of absorption as a function of pyruvate concentration.

### Signal stability

The final product of the DNPH-Glo II method is the hydrazone adduct. A pyruvate concentration of 0.5 mM was used to assess the stability of the resulting-colored product. The absorbance was measured at a wavelength of 430 nm at specific time intervals: 15 min, 30 min, 1 h, 2 h, 3 h, 4 h, 5 h, 24 h, 48 h, 72 h, 96 h, and 120 h. All measurements were taken at a controlled temperature of 25°C. The storage temperature was 25°C.

### Linearity and sensitivity

The sensitivity and linearity of the current method were evaluated by testing a range of Glo II activities from 0.05 to 100 units per liter (Ul^−1^). The study compared the method's linearity with the UV assay [14] using statistical analyses, specifically linear regression analysis with Qi Macros for Microsoft Excel 2020 (version 2020.07.15, QiMacros, Know Ware International, Denver, USA). In addition, the sensitivity of the DNPH-Glo II method was determined by calculating the limits of quantitation (LOQ) and detection (LOD) [15].

### Matrix effect

The matrix effect describes the influence of sample components other than the target analyte, on the accuracy of an analytical assay. In the DNPH-Glo II assay context, carbonyl-containing compounds present in biological samples could potentially interfere with the measurement of Glo II activity. A control test tube was therefore incorporated into the experimental design to control for the matrix effect in the DNPH-Glo II assay. This control tube contains all assay components except for the Glo II enzyme, allowing for the measurement of absorbance solely due to interfering compounds in the sample. By subtracting the absorbance of the control from the absorbance of the sample, the contribution of interfering compounds is effectively removed, ensuring that the remaining absorbance accurately reflects the unreacted substrates and provides a reliable measure of Glo II activity. This subtraction method effectively eliminates any potential bias introduced by the matrix effect, enhancing the precision and accuracy of the modified DNPH-Glo II assay.

### Validation

To assess the agreement between the DNPH-Glo II method and the established UV-GLO II method [14], a Bland–Altman analysis [16] was conducted. A series of Glo II-activity levels (0.5–100 U/l) were generated using the spinal cord Glo II enzyme [12]. Statistical analyses were performed using Qi Macros for Microsoft Excel 2020 (Know Ware International, Denver, CO, USA).

The Bland–Altman plot, a graphical representation of the agreement between two methods, was employed to evaluate the limitations of agreement between the DNPH-Glo II and UV-Glo II methods. This plot visualizes the differences between the two methods’ results, with the *y*-axis representing the absolute difference between the measurements ([B − A]) and the *x*-axis representing the average of the two measurements ([A + B]/2). According to Bland and Altman [15], 95% of the data points should fall within 1.96 standard deviations of the mean difference. This analysis allows for a visual assessment of the agreement between the methods, considering the distribution of differences and the assumptions of normality.

## Results and discussion

**Scheme 1.**
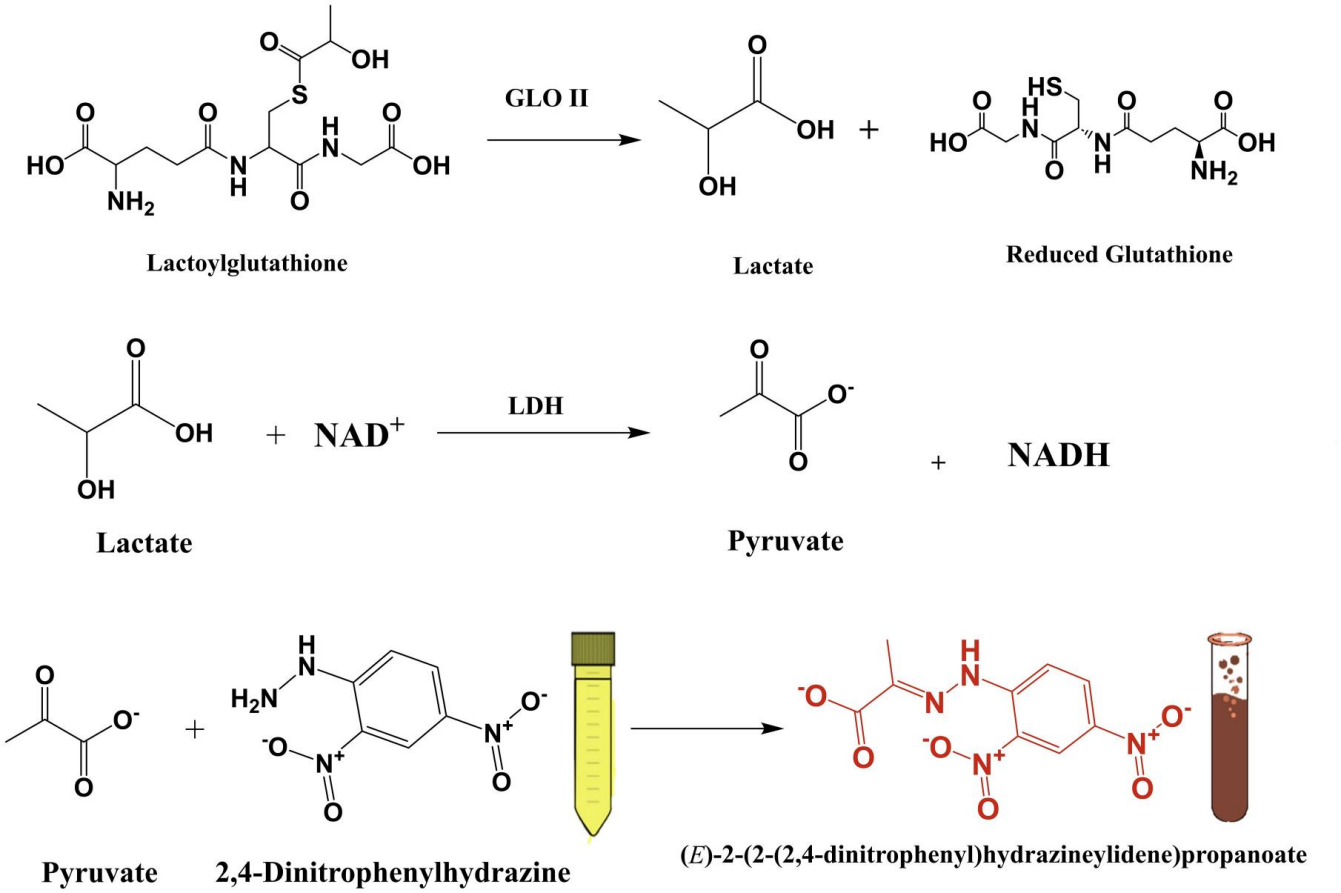
The Glo II enzymatic reaction. The D-lactate produced by the Glo II-catalyzed reaction is further converted to pyruvate by lactate dehydrogenase. The pyruvate reacts with the 2,4-DNPH reagent to form a brown-colored hydrazone product in the presence of sodium hydroxide.

This study used 2,4-DNPH to measure Glo II enzymatic activity. The principle behind this method is the formation of a colored hydrazone complex between 2,4-DNPH and the pyruvate that resulted from the indirect activity of the Glo II reaction. Glo II catalyzes the hydrolysis of SLG to produce D-lactate and reduced GSH. In the current assay, the D-lactate produced by the Glo II-catalyzed reaction is further converted to pyruvate by adding lactate dehydrogenase. The pyruvate then reacts with the 2,4-DNPH reagent to form a brown-colored hydrazone product, as shown in Scheme 1. The absorbance of this complex can be measured at 430 nm wavelength, which allows for the quantification of the Glo II activity.

**Figure 2:**
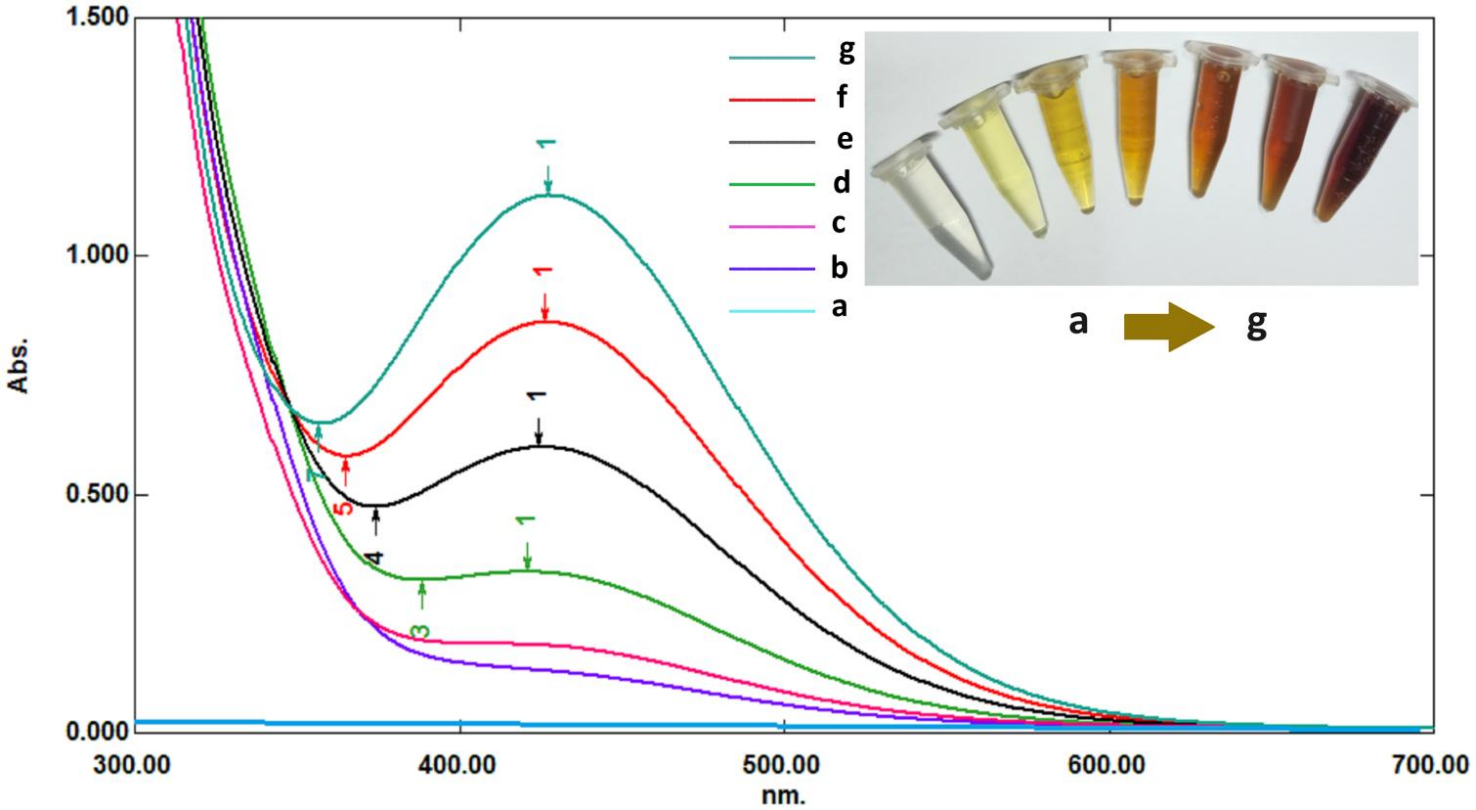
The spectral properties of the brown-colored hydrazone product were linked with the pyruvate concentration produced by Glo II activity. The resulting product was measured spectrophotometrically at 430 nm. Values (a–g) correspond to the pyruvate concentration of 250, 200, 150, 100, 50, 25, and 5 µmole/l, respectively.

**Figure 3:**
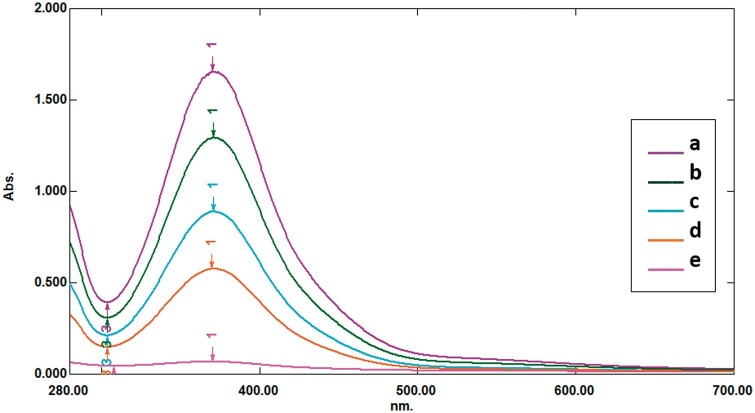
The spectral properties of the golden-colored guanidine-hydrochloride-enhanced hydrazone product were linked with the pyruvate concentration produced by Glo II activity. The resulting product was measured spectrophotometrically at 370 nm. Values (a–g) correspond to the pyruvate concentration of 250, 200, 150, 100, and 5 µmole/l, respectively.

As illustrated in Fig. 2, the color intensity and absorbance are directly correlated with the amount of lactate produced by Glo II activity. One unit of Glo II enzyme is defined as the amount of enzyme that catalyzes the production of one micromole of lactate per unit of time. Sodium hydroxide converts the adduct to a bright-brown end product, exhibiting strong absorbance at 430 nm. In an additional experiment, guanidine hydrochloride was used to clarify the color. Guanidine hydrochloride produces a golden color with a maximum absorbance of 370 nm, as shown in Fig. 3.

In all practical experiments, this study utilized sodium hydroxide, not guanidine hydrochloride, to convert the color to a bright brown end product because the absorbance at 430 nm was less affected by interferences.

The advantages of using the 2,4-DNPH method to measure Glo II activity include its relatively simple and cost-effective protocol and ability to quantitatively assess the enzyme’s activity. The assay can also be performed using a standard spectrophotometer, making it accessible to many laboratories. One key advantage of this method is that it indirectly measures Glo II activity by quantifying the pyruvate produced rather than directly measuring the hydrolysis of SLG. This can be useful in situations where the direct measurement of the substrate is challenging or less feasible.

### Signal stability

This study’s findings indicate that the colored compound exhibits exceptional stability at 25°C, as evidenced by the consistent absorbance of the brown-colored hydrazone product at 430 nm over three days. Specifically, the measurements revealed a negligible change in absorbance during this timeframe. However, a slight decrease in absorbance was observed on the fourth day (3%) and fifth day (7%), suggesting a gradual compound degradation over a very extended period.

### Linearity

The DNPH-Glo II method was linear across the range 0.005–500 µM pyruvate (Pearson’s *r *=* *0.997; Fig. 3).

### Sensitivity

**Figure 4:**
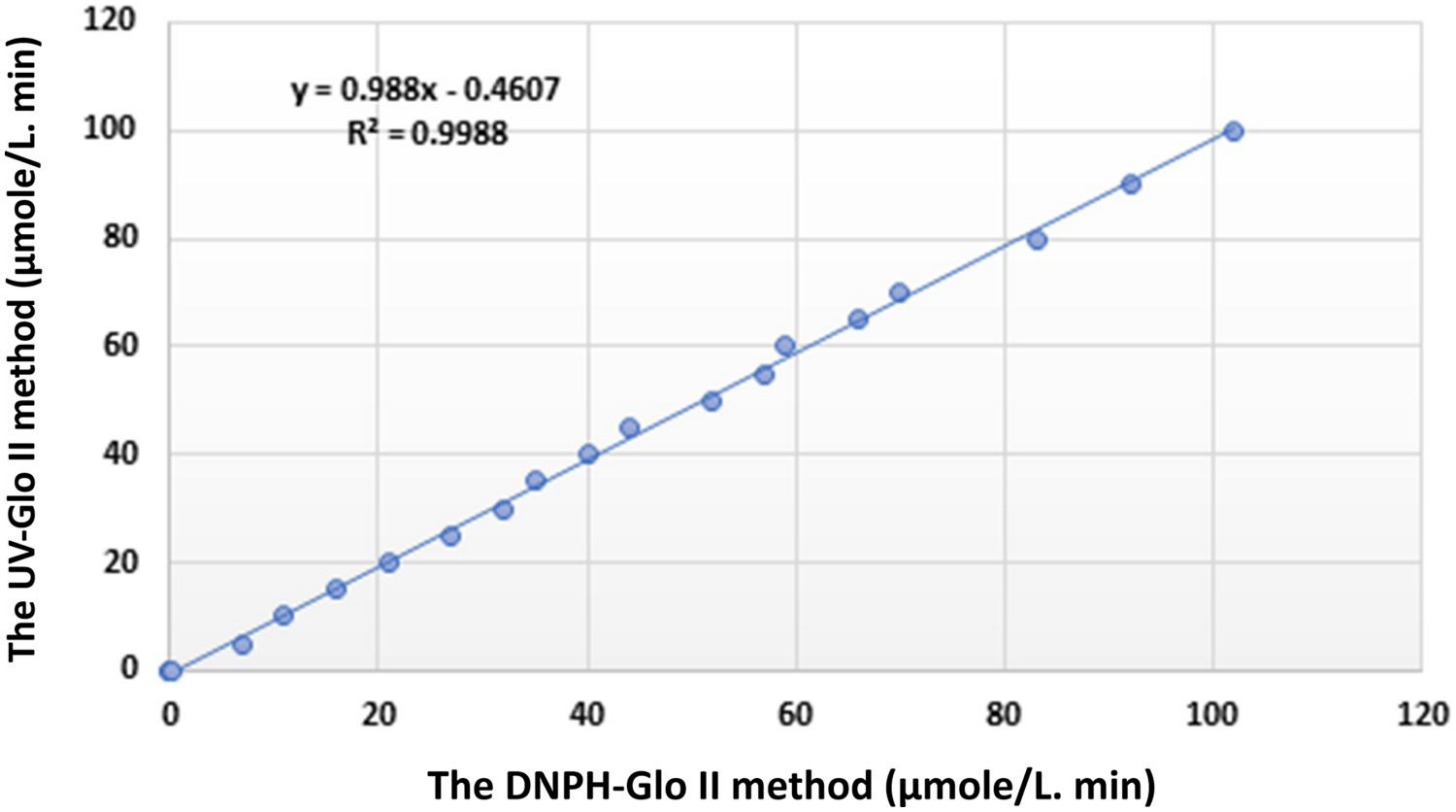
The protocol’s linearity was validated by plotting a straight line through the results of both the DNPH-GLO II assay and the UV-Glo II assay for various dilutions of Glo II activity. The range of Glo II activity tested was between 0.01 and 100 U/l. The enzymatic solution was incubated at 37°C for 30 min. The results presented are the mean of three independent experiments.

The DNPH-Glo II method was compared with the UV-Glo II method across the 0.01–100 Ul^−1^ Glo II enzyme activity range. The results show good agreement between the two methods (Pearson’s r = 0.998; see Fig. 4). The low values for LOD (0.004 Ul^−1^) and LOQ (0.012 Ul^−1^) demonstrate the remarkable sensitivity of the DNPH-Glo II method.

### Accuracy and selectivity of the DNPH-Glo II assay

Table 2 shows the results of a detailed study on interference, demonstrating the effectiveness of the proposed DNPH-Glo II assay in measuring Glo II activity in the presence of different potentially interfering biochemical substances. Several experiments were carried out to evaluate the assay's resistance to interference. For this purpose, 1 ml of a Glo II solution (40 Ul^−1^) was mixed with 1 ml of individual biochemical solutions. Glo II activity was then adjusted to 20 Ul^−1^ using the UV method, as previously described.

**Table 2.** The correlation between relative percentage errors and interfering biomolecules in the measurement of Glo II activity using the DNPH-Glo II assay.

Flask no.	Added Glo II	Found Glo II	Relative error^a^
	(U/l)	(U/l)	(%)
1	20	20	0.00
2	20	20.7	3.5
3	20	20.5	2.5
4	20	20.7	3.5
5	20	19.8	1.0
6	20	19.5	2.5
7	20	20.5	2.5
8	20	20.6	3.0

a Relative error is the ratio of the absolute error of the measurement to the true value of the measurement.

The interference study was carefully planned, involving eight flasks containing a unique combination of potentially interfering biomolecules. Flask 1 was a control, containing only phosphate-buffered saline at pH 7.4. Flask 2 contained a mixture of three monosaccharides (1 mM ribose, xylose, and glucose) to assess their impact on Glo II activity. To evaluate their interference, flask 3 contained a blend of four amino acids (1 mM histidine, glutamic acid, glutamine, and methionine). Flask 4 contained 1% (w/v) bovine serum albumin (BSA), a commonly used protein in biochemical assays, to investigate potential interference from proteins.

Flask 5 examined the effects of two small molecules, 100 µM uric acid and 100 µM sodium linoleate, on Glo II activity. Flask 6 contained 1 mM ethylenediaminetetraacetic acid, a metal chelator, to assess its potential impact on the assay. Flask 7 included a Protease Inhibitor Cocktail (10 μl/ml) to assess the effect of protease inhibition on Glo II activity. Finally, Flask 8 contained 0.1% (w/v) sodium dodecyl sulfate, a detergent commonly used in biochemical applications, to investigate its potential for interference or enzyme inhibition.

The results of this comprehensive interference study are presented in Table 1, showing the relative percentage errors associated with each added biochemical combination. This detailed presentation provides a comprehensive understanding of the proposed DNPH-Glo II assay’s performance in the presence of various potentially interfering species.

### Application to human-serum studies

The DNPH-Glo II assay was further utilized to evaluate Glo II activity in human serum. This study included 100 male students from the College of Science at the University of Babylon in Iraq. The participants had an average age of 22.0 ± 2 years and an average body mass index of 23.14 ± 1.5 kg/m^2^. All volunteers provided informed written consent after a clearly explanation of the study's objectives. The participants were divided into two groups: smokers and non-smokers. The non-smoker group consisted of individuals with no history of smoking, while the smokers had been smoking an average of 20 ± 5 cigarettes per day for over two years. All participants were non-alcoholics and free from chronic diseases. The institutional ethics committee approved the study. Following an overnight fast, 5 ml of venous blood was collected into heparin from each participant. The blood was centrifuged at 3000 rpm for 10 min to separate the plasma from the erythrocytes. The resulting clear serum was then used to determine Glo II activity.

The data presented in Table 3 indicate that serum Glo II activity is significantly higher in smokers compared to non-smokers (*P* < .05). This result indicates that tobacco smoking is linked to increased Glo II activity. Smoking introduces harmful substances into the body, including dicarbonyl metabolites [17]. Prolonged exposure to tobacco smoke can upset the equilibrium between the production of reactive oxygen species and the body’s capacity to counteract them [18]. This disruption increases Glo II activity as the body strives to counteract the harmful effects of the heightened levels of dicarbonyl compounds. The increased Glo II activity observed in smokers is likely a compensatory response to the elevated harmful dicarbonyl compounds concentration introduced by tobacco smoking.

**Table 3.** Comparison of Glo II activity between tobacco smokers and non-smokers.

	Non-smokers	Smokers	*P*-value
Glo II (U/l)	66 ± 12	87 ± 21	.005

### Reproducibility

This study examined the activity of Glo II in serum samples to evaluate the precision and accuracy of the experimental protocol. A serum sample was divided into 100 fractions and stored at −80°C until assessment. The results presented in Table 4 indicate that the Glo II activity measured using the DNPH-Glo II method was consistent with the levels obtained using the UV-Glo II method. This suggests that the two methods provide comparable results for assessing Glo II activity. Furthermore, the intra-day precision of the assay was satisfactory, with relative standard deviation (RSD%) values ranging from 2.11% to 3.97% (Table 4). This means that the repeated measurements within the same day showed a high degree of consistency.

**Table 4.** Comparison of Glo II (U/l) activities in serum using the UV-Glo II and DNPH-Glo II methods.

The UV-Glo II method				The DNPH-Glo II method			
Intra-day ±SD	RSD%	Inter-day ±SD	RSD%	Intra-day ±SD	RSD%	Inter-day ±SD	RSD%
71 ± 1.5	2.11	68 ± 2.7	3.97	73 ± 2.7	3.69	70 ± 3.3	4.71

The assessment of the UV-Glo II method’s inter-day precision showed satisfactory results, with RSD% values ranging from 3.69% to 4.71% (Table 4). This indicates a high level of reproducibility in the measurements across different days. These findings confirm the accuracy and precision of the assay under varying experimental conditions. The low RSD% values for intra-day and inter-day precision demonstrate that the DNPH-Glo II method is accurate and precise for assessing Glo II activity in serum samples.

### Validation and method comparison

**Figure 5:**
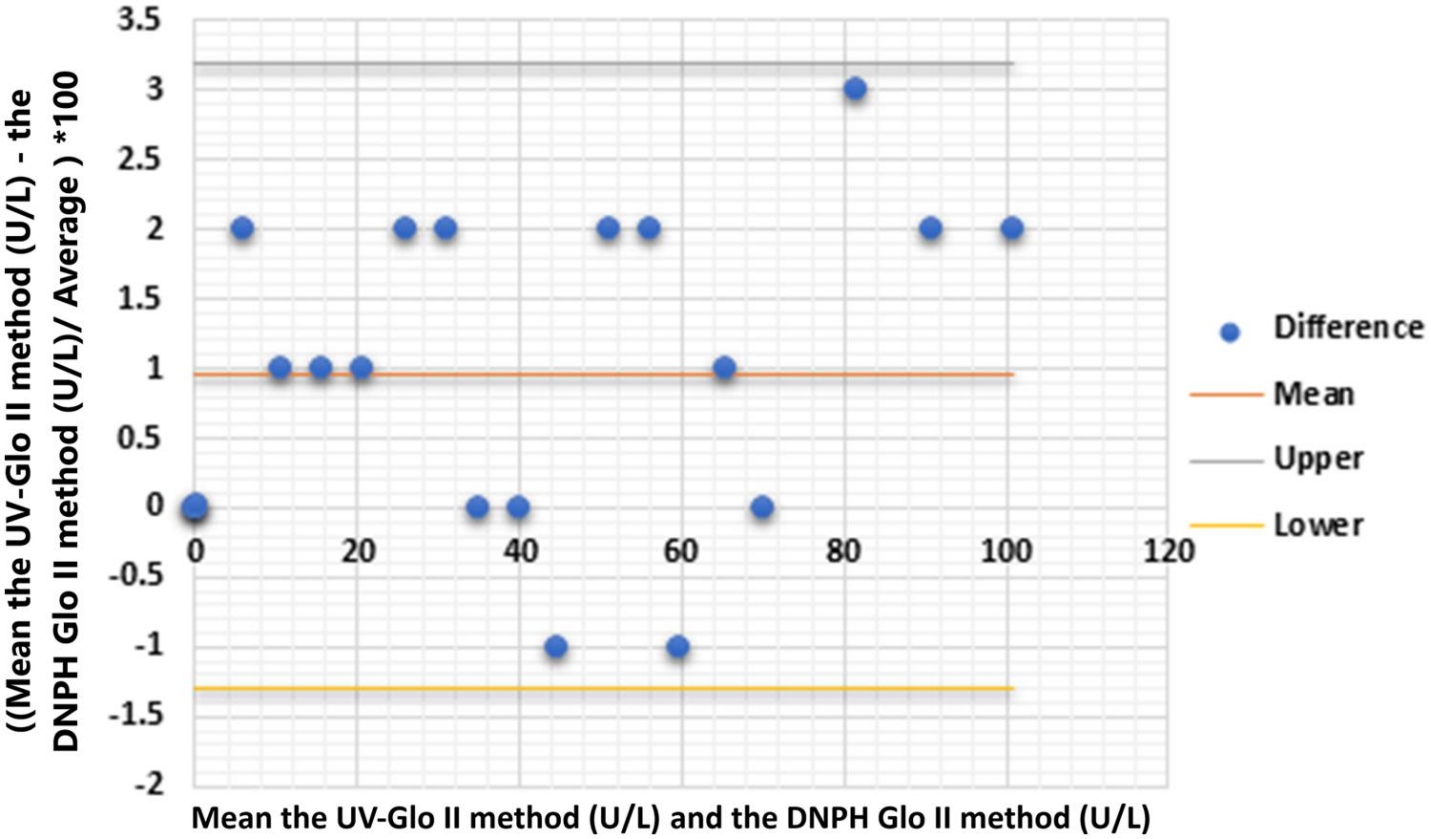
The Bland–Altman plot shows the average relative bias and relative differences in Glo II activity (U/l), measured by both the DNPH-Glo II assay and UV method. The Glo II activity levels range from 0.1 to 100 U/l. After a 30-min incubation of the enzyme solution at 37°C, the results were calculated as the average of three separate experiments.

This study compared the performance of the DNPH-Glo II assay and the UV method for measuring Glo II activity in serum samples using the Bland–Altman approach [16]. The serum Glo II activity ranged from 0.1 to 100 U/l and was utilized to compare the two protocols. The Bland–Altman plot (Fig. 5) displays the mean relative bias and the relative differences between the DNPH-Glo II and UV methods. The correlation between the two methods was 99.5%, indicating an extremely strong agreement.

This result suggests that the DNPH-Glo II assay is almost as precise as the reference standard UV method for evaluating Glo II activity in serum samples. Overall, the findings indicate that the DNPH-Glo II assay is a dependable and accurate alternative to the UV method for measuring Glo II activity in serum, with a very high level of agreement between the two techniques.

## Conclusions

A novel protocol for measuring Glo II activity has been presented, offering a straightforward and efficient approach. This method can accurately assess Glo II activity in diverse biological samples, even in the presence of high concentrations of interfering substances. The protocol is based on the principle that increased absorbance is directly proportional to elevated Glo II activity. Notably, the DNPH-Glo II assay resists interferences caused by test amino acids, carbohydrates, and proteins, ensuring reliable results. The assay’s sensitivity is attributed to the chemical reagent 2,4-DNPH, which enables the detection of Glo II activity at low substrate concentrations. Overall, the 2,4-DNPH-based assay is a valuable tool for measuring Glo II activity, offering a simple and accessible solution for research and diagnostic applications.

While the DNPH-Glo II method offers a sensitive and specific approach for quantifying Glo II activity, it has limitations. Compared to the DTNB assay, it is more time-consuming and requires additional steps and reagents. However, it is essential to acknowledge the strengths of the DNPH-Glo II method, including its ability to detect Glo II activity and its high sensitivity specifically.

## Data Availability

The authors declare that all data supporting the findings of this study can be found within the article. Additional data supporting the findings of this study are available from the corresponding author upon request.

## References

[1] Braun BC , Müller K. Role of glyoxalase I and II in somatic and spermatogenic testicular cells during the postnatal development of the domestic cat. Theriogenology 2023;197:10–5. 36462331 10.1016/j.theriogenology.2022.11.028

[2] Farrera DO , Galligan JJ. The human glyoxalase gene family in health and disease. Chem Res Toxicol 2022;35:1766–76. 36048613 10.1021/acs.chemrestox.2c00182 PMC10013676

[3] Roberta G , Emiliano L, Emanuele F. et al Protein–protein interactions of human glyoxalase II: findings of a reliable docking protocol. Org Biomol Chem 2018;16:5167–77. 10.1039/C8OB01194J 29971290

[4] Alhujaily M. Glyoxalase system in breast and ovarian cancers: role of MEK/ERK/SMAD1 pathway. Biomolecules 2024;14:584. 10.3390/biom14050584 38785990 PMC11117840

[5] Scirè A , Cianfruglia L, Minnelli C. et al Glyoxalase 2: towards a broader view of the second player of the glyoxalase system. Antioxidants 2022;11:2131. 10.3390/antiox11112131 36358501 PMC9686547

[6] Christoph TT , Elisabeth L, Katrin B. et al Loss of glyoxalase 2 alters the glucose metabolism in zebrafish. Redox Biology 2022;59:102576. 10.1016/j.redox.2022.102576 36535130 PMC9792892

[7] Maura NL , Federica B, Mario S. Glyoxalase I assay as a possible tool for evaluation of biological activity of antioxidant-rich plant extracts. Plants 2023;12:1150. 10.3390/plants12051150 36904010 PMC10005046

[8] Oprea E , Cinteza D, Berteanu M. et al The relationship between alanerv^®^ consumption and erythrocytes' glyoxalases I and II activities and the level of some serum markers of carbonyl stress in post-acute stroke patients undergoing rehabilitation. Maedica (Bucur) 2013;8:249–55. 24371493 PMC3869113

[9] Arai M , Nihonmatsu-Kikuchi N, Itokawa M. et al Measurement of glyoxalase activities. Biochem Soc Trans 2014; 42:491–4. 10.1042/BST20140010 24646266

[10] Principato GB , Rosi G, Talesa V. et al L. Purification and characterization of two forms of glyoxalase II from the liver and brain of Wistar rats. Biochim Biophys Acta (BBA)-Protein Struct Mol Enzymol 1987;911:349–55. 10.1016/0167-4838(87)90076-8 3814608

[11] Ryan LD , Vestling CS. Rapid purification of lactate dehydrogenase from rat liver and hepatoma: a new approach. Arch Biochem Biophys 1974;160:279–84. 4364067 10.1016/s0003-9861(74)80035-4

[12] Skapare E , Konrade I, Liepinsh E. et al Glyoxalase 1 and glyoxalase 2 activities in blood and neuronal tissue samples from experimental animal models of obesity and type 2 diabetes mellitus. J Physiol Sci 2012;62:469–78. 22893478 10.1007/s12576-012-0224-9 PMC10717385

[13] Kummari R , Puja R, Bose K. Protein quantitation and detection. In: Bose K (ed.), Textbook on Cloning, Expression and Purification of Recombinant Proteins. Singapore: Springer Nature Singapore, 2022, 279–99.

[14] Vander Jagt DL , Han LP, Lehman CH. Kinetic evaluation of substrate specificity in the glyoxalase-I-catalyzed disproportionation of α-ketoaldehydes. Biochemistry 1972;11:3735–40. 5072200 10.1021/bi00770a011

[15] Nowak PM , Wietecha-Posłuszny R, Pawliszyn J. White analytical chemistry: an approach to reconcile the principles of green analytical chemistry and functionality. TrAC Trend Anal Chem 2021;138:116223.

[16] Karun KM , Puranik A. BA. plot: an R function for Bland-Altman analysis. Clin Epidemiol Global Health 2021;12:100831.

[17] Mure K , Tomono S, Mure M. et al The combination of cigarette smoking and alcohol consumption synergistically increases reactive carbonyl species in human male plasma. Int J Mol Sci 2021;22:9043. 34445749 10.3390/ijms22169043 PMC8396601

[18] Seo YS , Park JM, Kim JH. et al Cigarette smoke-induced reactive oxygen species formation: a concise review. Antioxidants 2023;12:1732. 37760035 10.3390/antiox12091732 PMC10525535

